# Talin, a Rap1 effector for integrin activation at the plasma membrane, also promotes Rap1 activity by disrupting sequestration of Rap1 by SHANK3

**DOI:** 10.1242/jcs.263595

**Published:** 2025-02-26

**Authors:** Zhongji Liao, Sanford J. Shattil

**Affiliations:** Department of Medicine, University of California, San Diego, La Jolla, CA 92093, USA

**Keywords:** Rap1, Talin, SHANK3, Endothelial cell, Optogenetics

## Abstract

Talin regulates the adhesion and migration of cells in part by promoting the affinity of integrins for extracellular matrix proteins, a process that in cells such as endothelial cells and platelets requires the direct interaction of talin with both the small GTPase Rap1 bound to GTP (Rap1–GTP) and the integrin β3 cytoplasmic tail. To study this process in more detail, we employed an optogenetic approach in living, immortalized endothelial cells to be able to regulate the interaction of talin with the plasma membrane. Previous studies identified talin as the Rap1–GTP effector for β3 integrin activation. Surprisingly, optogenetic recruitment of talin-1 (TLN1; herein referred to as talin) to the plasma membrane also led to the localized activation of Rap1 itself, apparently by talin competing for Rap1–GTP with SHANK3, a protein known to sequester Rap1–GTP and to block integrin activation. Rap1 activation by talin was localized to the cell periphery in suspension cells and within lamellipodia and pseudopodia in cells adherent to fibronectin. Thus, membrane-associated talin can play a dual role in regulating integrin function in endothelial cells: first, by releasing Rap1–GTP from its sequestration by SHANK3, and second, by serving as the relevant Rap1 effector for integrin activation.

## Teaser

**Highlighted Article:** Optogenetic recruitment of talin to the plasma membrane promotes Rap1 activity by freeing Rap1–GTP from sequestration by SHANK3, enabling the Rap1–GTP–talin complex to activate integrins.

## INTRODUCTION

Talin is a ∼270 kDa cytoskeletal protein that consists of an N-terminal FERM head domain and a C-terminal rod domain. It plays critical roles in cell biology, in part by regulating the function of integrin adhesion receptors (Calderwood, 2004). This is exemplified by its function in platelets and endothelial cells where it is essential for regulating the affinity of integrins, including αIIbβ3 (consisting of ITGA2B and ITGB3) in platelets and αVβ3 (ITGAV and ITGB3) in endothelial cells, for their respective adhesive matrix ligands (Nieswandt et al., 2007; Petrich et al., 2007; Pulous et al., 2019; Tadokoro et al., 2003). When cells become activated, talin is recruited to the inner surface of the plasma membrane, where its head domain can interact with both the small GTPase Rap1 and the β3 integrin cytoplasmic tail (Calderwood et al., 1999; Camp et al., 2018; Gingras et al., 2019). Like other Ras-like guanine nucleotide-binding proteins, Rap1 can cycle between inactive GDP-bound (Rap1–GDP) and active GTP-bound (Rap1–GTP) states through the actions of one or more guanine nucleotide exchange factors and GTPase-activating proteins (Bos et al., 2007). In current models of β3 integrin activation, cell activation leads to the conversion of membrane-anchored Rap1–GDP to Rap1–GTP, with talin then functioning as the relevant Rap1 effector for integrin affinity regulation (Sun et al., 2022). However, gaps remain in our knowledge about this process, including the exact sequence of events following talin recruitment to the plasma membrane.

In the present study, we focused on this particular sequence of events by utilizing an optogenetic technique to rapidly enforce the interaction of full-length talin-1 (TLN1; hereafter referred to as talin) with the plasma membrane of endothelial cells in response to 450 nm (blue) light. We confirm that talin recruitment to the plasma membrane triggers β3 integrin activation in a process that requires interaction of the talin head domain with both Rap1 and the integrin β3 cytoplasmic tail (Liao et al., 2021). Unexpectedly, we found that talin interaction with the plasma membrane also promotes the availability of active Rap1–GTP itself. By pursuing the mechanism of this process, we conclude that not only is talin a direct effector of Rap1 in integrin signaling, but it also regulates Rap1–GTP activity at the plasma membrane.

## RESULTS

### Translocation of talin to the plasma membrane leads to activation of Rap1

**Fig. 1.**
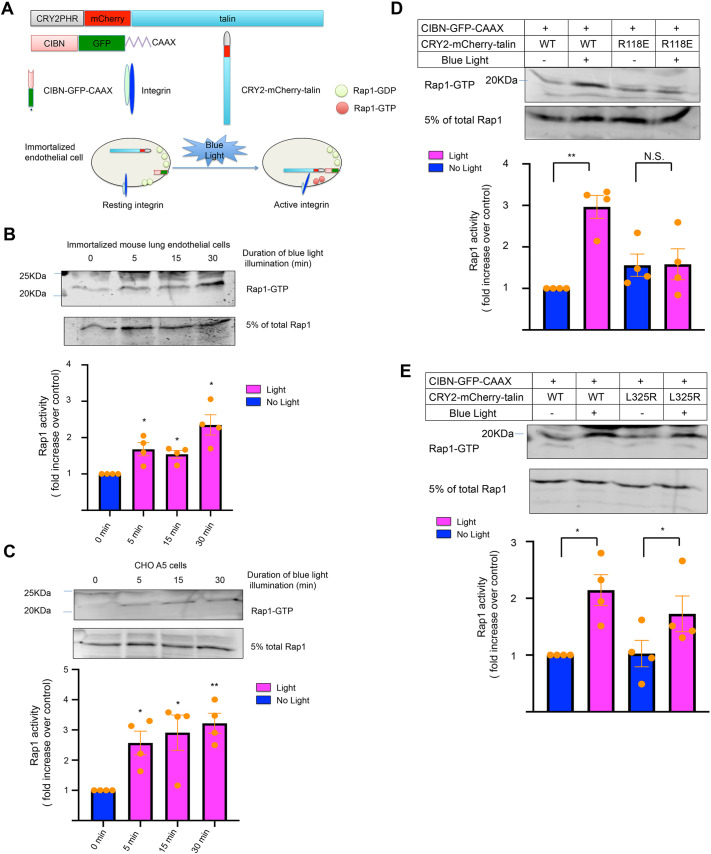
**Optogenetic recruitment of talin to the plasma membrane of endothelial cells leads to activation of Rap1.** (A) Schematic representation of the optogenetic constructs CIBN–GFP–CAAX and CRY2–mCherry–talin expressed in immortalized mouse lung endothelial cells. The CIBN moiety is anchored to the plasma membrane and recruits CRY2–mCherry–talin upon exposure of the cells to 450 nm (blue) light. Previous work has shown that such recruitment leads to activation of integrin αVβ3 (Liao et al., 2021). Rap1 activation, the transition from Rap1–GDP to Rap1–GTP, can also be monitored during this process. (B) Time course of Rap1 activation in endothelial cells in response to blue light illumination. Rap1–GTP was selectively pulled down using agarose beads loaded with the Rap-binding domain of RalGDS and detected using an anti-Rap1 antibody. Upper panel: representative western blots of the Rap1–GTP pull-down assay and total Rap1 in whole-cell lysates (input: 5%). Lower panel: quantitative analysis of Rap1–GTP. The ratio of Rap1–GTP to total Rap1 was calculated and normalized to that observed at time zero when the cells were maintained in the dark. Data represent means±s.e.m. of four experiments (*P<0.05; paired two-tailed Student's *t*-test). (C) Time course of Rap1 activation in response to blue light in A5 CHO cells stably expressing integrin αIIbβ3, CIBN–GFP–CAAX and CRY2–mCherry–talin. Data represent means±s.e.m. of four experiments (*P<0.05; **P<0.01; paired two-tailed Student's *t*-test). (D) Recruitment to the plasma membrane of a CRY2–mCherry–talin mutant (R118E) that cannot interact with Rap1 fails to activate Rap1 in A5 CHO cells. Data represent means±s.e.m. of four experiments (N.S., not significant; **P<0.01; paired two-tailed Student's *t*-test). (E) Expression of a CRY2–mCherry–talin mutant (L325R) defective in activating integrins still enables Rap1 activation in these cells upon recruitment of CRY2–mCherry–talin to the plasma membrane in A5 CHO cells. Data represent means±s.e.m. of four experiments (*P<0.05; paired two-tailed Student's *t*-test).

We utilized an optogenetic system (Liao et al., 2021) to rapidly enforce the recruitment of full-length talin to the inner surface of the plasma membranes of immortalized murine lung endothelial cells that normally express integrin αVβ3, or A5 Chinese hamster ovary (CHO) cells that express recombinant integrin αIIbβ3. When the constructs encoding CRY2–mCherry–talin [containing the full-length sequence of mouse talin-1 (TLN1)] and CIBN–GFP–CAAX were stably expressed in these cells in the absence of 450 nm light, CRY2–mCherry–talin was located diffusely within the cells, whereas CIBN–GFP–CAAX was membrane anchored (Liao et al., 2021). However, as depicted schematically in Fig. 1A, the CRY2 and CIBN moieties can interact upon exposure to 450 nm light, thereby recruiting CRY2–mCherry–talin to the inner surface of the plasma membrane, where it would presumably be in proximity to membrane-anchored Rap1. Consistent with this idea, our previous study showed that optogenetic recruitment of talin to the plasma membrane triggers αVβ3 activation, as assessed by fibrinogen binding, a process known to be dependent on Rap1 (Liao et al., 2021). To monitor Rap1 activity in this optogenetic system, agarose beads containing the Rap-binding domain (RBD) of RalGDS were used to pull down Rap1–GTP from cell lysates. Surprisingly, translocation of CRY2–mCherry–talin to the plasma membrane triggered Rap1 activation (Fig. 1B; *P<*0.05 for 5, 15 and 30 min of illumination), and the level of Rap1 activation was similar to that observed when the cells were stimulated with basic fibroblast growth factor (bFGF, also known as FGF2) (Fig. S1A). This effect of membrane recruitment of talin on Rap1 activity was not unique to endothelial cells because it was also observed in the A5 CHO cell system (Fig. 1C; *P*<0.05 for 5 and 15 min of illumination; *P*<0.01 for 30 min of illumination), but not in cells expressing CIBN–GFP–CAAX and mCherry–talin (instead of CRY2–mCherry–talin) (Fig. S1B). Thus, recruitment of talin to the plasma membrane leads to increased Rap1 activity.

### Rap1 activation by talin requires their direct interaction

In platelets and A5 CHO cells, the direct interaction of Rap1 with the talin head F1 subdomain is required for β3 integrin activation, and this interaction can be prevented by introducing a mutation (R118E) into talin F1 (Lagarrigue et al., 2020; Liao et al., 2021). When CRY2–mCherry–talin (R118E) and CIBN–GFP–CAAX were co-expressed in A5 CHO cells, optogenetic recruitment of this talin mutant to the plasma membrane failed to activate Rap1 (Fig. 1D). This suggests that Rap1 activation in response to talin translocation to the plasma membrane requires the direct interaction of Rap1 with talin.

Next, we examined whether Rap1 activation subsequent to talin translocation to the plasma membrane is simply a consequence of integrin activation and ‘outside-in’ signaling through the active integrin. To investigate this possibility, we used a CRY2–mCherry–talin (L325R) mutant that can still bind to the distal portion of the β3 integrin cytoplasmic tail but cannot activate the integrin to bind extracellular ligands because membrane-proximal interactions of the mutant are impaired (Wegener et al., 2007; Liao et al., 2021). Despite this, optogenetic membrane recruitment of this talin mutant still led to activation of Rap1 (Fig. 1E; *P*<0.05). Thus, integrin activation and subsequent outside-in integrin signaling are not required for talin-mediated Rap1 activation at the plasma membrane.

### Translocation of talin to the plasma membrane promotes localization of active Rap1 to cell edges

**Fig. 2.**
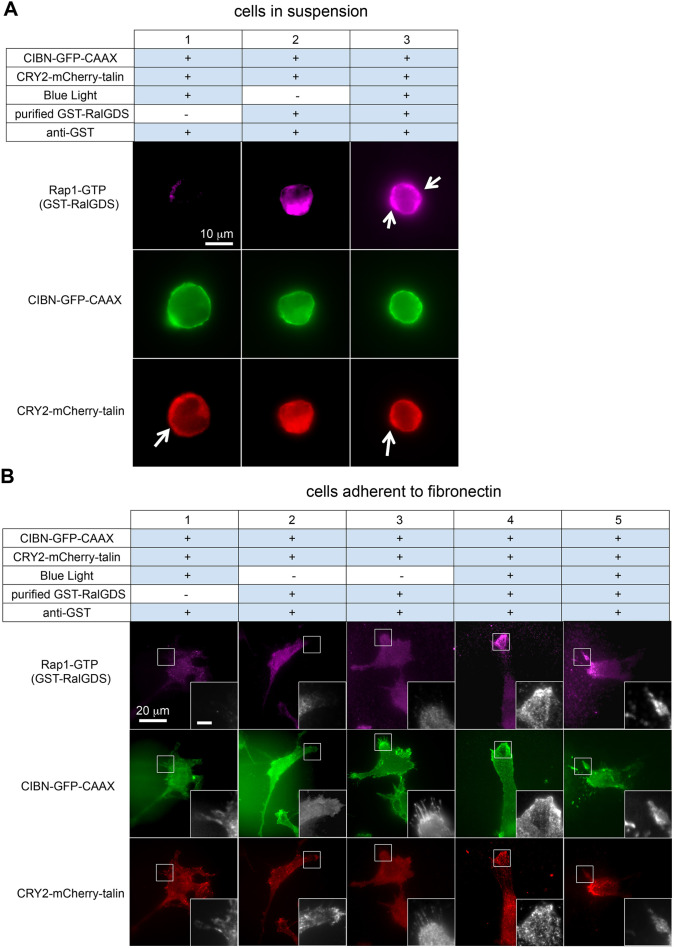
**Optogenetic recruitment of talin to the plasma membrane promotes active Rap1 localization to cell edges.** (A) Optogenetic recruitment of talin to the plasma membrane promotes active Rap1 localization to the cell periphery in suspended cells. Immortalized murine endothelial cells in suspension expressing CIBN–GFP–CAAX and CRY2–mCherry–talin were illuminated using blue light for 30 min before Rap1–GTP was detected *in situ* as described in the Materials and Methods. Samples without GST–RalGDS were used as a control (column 1). White arrows indicate enrichment of active Rap1 and CRY2–mCherry–talin at the cell periphery, the former only in response to blue light. Scale bar: 10 µm. (B) Optogenetic recruitment of talin to the plasma membrane enriches active Rap1 localization at cell protrusions in adherent cells. Immortalized murine endothelial cells expressing CIBN–GFP–CAAX and CRY2–mCherry–talin were plated on fibronectin-coated coverslips for 30 min before being illuminated with blue light or maintained in the dark for 30 min. Samples were fixed and *in situ* Rap1–GTP assay was performed as described in the Materials and Methods. Samples without GST–RalGDS incubation were used as a negative control and are shown in column 1. In these representative images, cell protrusions are highlighted by the small box in the main panel and presented as magnified insets on the bottom right of each image. Blue light illumination induces active Rap1 localization on cell lamellipodium-like protrusions (column 4) and pseudopodium-like protrusions (column 5). Note that such signal enrichment was not seen in cells maintained in the dark (columns 2 and 3) despite the formation of cell protrusions. Scale bars: 20 µm (main panel); 1 µm (inset). Images in A,B are representative of three independent experiments.

Rap1 regulates angiogenic responses of endothelial cells, including adhesion, proliferation and migration (Chrzanowska-Wodnicka, 2010). Moreover, active Rap1–GTP has been found to localize at the leading edges of migrating endothelial cells using GST–RalGDS as a reporter (Fujita et al., 2005). Therefore, to study the subcellular localization of active Rap1 in response to optogenetic recruitment of talin to the plasma membrane, we used GST–RalGDS in fluorescence microscopy (Fig. 2). Upon blue light illumination, there was an overall increase in GST–RalGDS staining in the cells, and a marked enrichment of the signal at the periphery of cells maintained in suspension (Fig. 2A). Furthermore, when the cells were plated on fibronectin and allowed to spread, blue light illumination increased the GST–RalGDS signal within lamellipodia (Fig. 2B, column 4) and pseudopodia (Fig. 2B, column 5). In contrast, cells maintained in the dark failed to localize active Rap1 to the cell periphery (Fig. 2B, columns 2 and 3). Taken together, these results support the idea that talin translocation to the plasma membrane promotes the localized activation of Rap1.

### A potential mechanism of Rap1 activation by talin

**Fig. 3.**
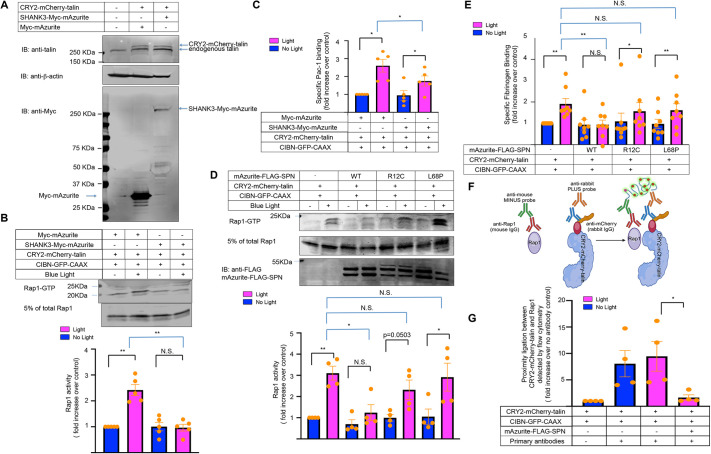
**Overexpression of SHANK3 blocks Rap1 activation induced by talin recruitment to the plasma membrane.** (A–C) SHANK3 tagged with Myc–mAzurite was transfected into A5 CHO cells stably expressing CIBN–GFP–CAAX and CRY2–mCherry–talin. Cells triple positive for mAzurite, GFP and mCherry were sorted by flow cytometry. Cells expressing Myc–mAzurite without SHANK3 served as a control. (A) Western blot analysis of SHANK3–Myc–mAzurite expression in these cells. β-actin served as a loading control. SHANK3 overexpression did not affect the levels of CRY2–mCherry–talin. The images represent two independent experiments. (B) SHANK3 overexpression inhibits Rap1 activation in response to the optogenetic recruitment of talin to the plasma membrane. Data represent means±s.e.m. of five experiments (N.S., not significant; **P<0.01; paired two-tailed Student's *t*-test). (C) SHANK3 blunts activation of integrin αIIbβ3 in response to the optogenetic recruitment of talin to the plasma membrane. Activation of integrin αIIbβ3 was monitored by flow cytometry using the PAC-1 antibody and expressed as the fold increase relative to that observed when cells were maintained in the dark. Data represent means±s.e.m. of five experiments (*P<0.05; paired two-tailed Student's *t*-test). (D–G) Lentiviruses encoding the WT SPN domain of SHANK3 [mAzurite–FLAG–SPN (WT)], the R12C SPN mutant [mAzurite–FLAG–SPN (R12C)] or the L68P SPN mutant [Myc–mAzurite–FLAG–SPN (L68P)] were transduced into immortalized murine lung endothelial cells expressing CIBN–GFP–CAAX and CRY2–mCherry–talin. Cells infected with empty lentiviral vector served as controls. (D) WT SPN, but not R12C or L68P SPN, inhibits Rap1 activation following optogenetic recruitment of talin to the plasma membrane. Data represent mean±s.e.m. of four experiments (N.S., not significant; *P<0.05; paired two-tailed Student's *t*-test). (E) WT SPN, but not the R12C or L68P SPN mutants, inhibits specific fibrinogen binding to integrin αVβ3 upon optogenetic recruitment of talin to the plasma membrane. Data represent means±s.e.m. of eight experiments (N.S., not significant; *P<0.05; **P<0.01; paired two-tailed Student's *t*-test). (F,G) Duolink proximity ligation assay (PLA) was performed to examine the effects of SHANK3 SPN on the association of Rap1 with CRY2–mCherry–talin in endothelial cells. (F) Schematic representation of the Duolink PLA. Created in BioRender by Liao, Z., 2025. https://BioRender.com/m47c469. This figure was sublicensed under CC-BY 4.0 terms. (G) After 30 min of incubation at room temperature in the absence or presence of blue light illumination, cells were fixed, permeabilized and stained with rabbit anti-mCherry and mouse anti-Rap1 antibodies. Then, Duolink PLA flow cytometry was performed to assess the interaction between CRY2–mCherry–talin and Rap1. Cells kept in the dark and untreated with primary antibodies served as controls. Data represent means±s.e.m. of four experiments (*P<0.05; paired two-tailed Student's *t*-test).

Given that talin-mediated activation of Rap1 required the direct interaction between the two proteins upstream of integrin activation, our attention then focused on the potential mechanism. SH3 and multiple ankyrin repeat domains 3 (SHANK3) has been implicated in inhibiting integrin function in cancer cells by binding to and sequestering active Rap1–GTP through its N-terminal SPN domain, thus limiting Rap1–GTP bioavailability at the plasma membrane (Lilja et al., 2017). Although originally identified as a scaffold protein in the central nervous system and mutated in a spectrum of neuropsychiatric disorders (Naisbitt et al., 1999), we observed SHANK3 expression in murine lung endothelial cells (Fig. S2A). Short hairpin RNA (shRNA)-mediated silencing of SHANK3 in these cells led to a slightly elevated basal level of Rap1 activation and fibrinogen binding, which increased further upon light-induced translocation of talin to the plasma membrane (Fig. S2B,C). Moreover, overexpression of full-length SHANK3 in A5 CHO cells (Fig. 3A) inhibited both Rap1 and αIIbβ3 activation following optogenetic translocation of talin to the plasma membrane (Fig. 3B, *P*<0.01; Fig. 3C, *P*<0.05, between control cells and cells expressing SHANK3–Myc–mAzurite).

Given the reported role of the SHANK3 SPN domain in interacting with Rap1–GTP, we overexpressed the fluorescently labeled, wild-type (WT) SPN domain (mAzurite–FLAG–WT SPN) in immortalized mouse endothelial cells co-expressing CRY2–mCherry–talin and CIBN–GFP–CAAX. Overexpression of mAzurite–FLAG–WT SPN blocked both optogenetic activation of Rap1 (Fig. 3D, *P*<0.05) and activation of αVβ3, the latter monitored by fibrinogen binding (Fig. 3E, *P*<0.01). In sharp contrast, two autism spectrum disorder-related SHANK3 variants (R12C and L68P) within the SPN domain that are unable to bind to Rap1–GTP (Lilja et al., 2017; Mameza et al., 2013) failed to block activation of Rap1 or αVβ3 in response to optogenetic recruitment of talin to the plasma membrane of endothelial cells (Fig. 3D,E). Thus, SHANK3 might be able to negatively regulate talin-mediated activation of Rap1 through its direct interaction with and sequestering of Rap1–GTP.

In a model in which SHANK3 and talin might compete for Rap1–GTP binding, SHANK3 might be expected to reduce talin-dependent integrin activation (Atherton and Ballestrem, 2017). To determine whether SHANK3 can interfere with the interaction between talin and Rap1 in endothelial cells, the interaction between CRY2–mCherry–talin and Rap1 was assessed by flow cytometry using the Duolink proximity ligation assay (PLA) (Leuchowius et al., 2009) (Fig. 3F,G; Fig. S3). Optogenetic recruitment of CRY2–mCherry–talin to the plasma membrane increased its interaction with Rap1 in this assay by ∼10%. In contrast, overexpression of the WT SHANK3 SPN domain (mAzurite–FLAG–WT SPN) reduced the interaction between Rap1 and CRY2–mCherry–talin by approximately 70% (Fig. 3G, *P*<0.05). These results suggest that SHANK3 and talin indeed compete for interaction with Rap1–GTP at the plasma membrane.

### Translocation of talin to the plasma membrane relieves the sequestration of Rap1–GTP by SHANK3

**Fig. 4.**
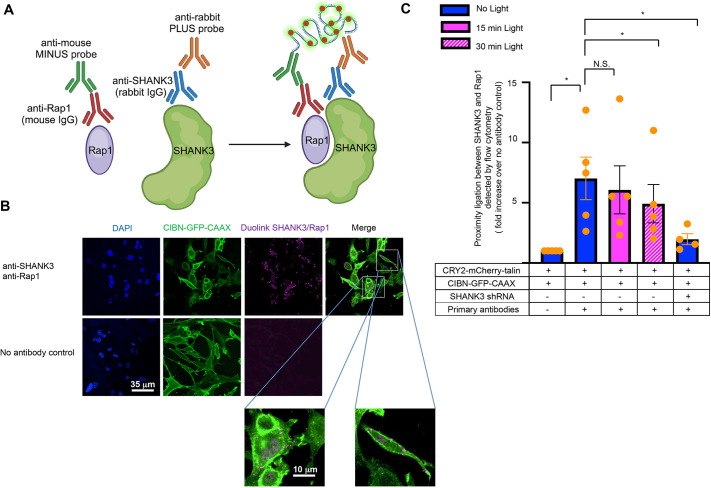
**Optogenetic recruitment of talin to the plasma membrane impairs Rap1 interaction with SHANK3.** (A) Schematic representation of the Duolink proximity ligation assay (PLA). Cells were fixed, permeabilized and stained with rabbit anti-SHANK3 and mouse anti-Rap1 antibodies before Duolink PLA was performed to assess the proximity of SHANK3 to Rap1. Created in BioRender by Liao, Z., 2025. https://BioRender.com/b40n281. This figure was sublicensed under CC-BY 4.0 terms. (B) Immortalized murine lung endothelial cells expressing CRY2–mCherry–talin and CIBN–GFP–CAAX were plated on fibrinogen overnight, fixed, permeabilized and stained with anti-SHANK3 and anti-Rap1 antibodies. PLA was performed to evaluate colocalization of endogenous SHANK3 and Rap1. Cell nuclei were counterstained with DAPI and cells were imaged by confocal microscopy. Cells kept in the dark and untreated with primary antibodies served as controls. Two areas within the images with merged signals for PLA and CIBN–GFP–CAAX are highlighted with boxes and presented as magnified insets on the bottom. PLA signals were observed within the cytoplasm (inset on the left) and on the plasma membrane (inset on the right). Scale bars: 35 µm (main panel); 10 µm (inset). Images represent two independent experiments. (C) Immortalized murine lung endothelial cells in suspension were either kept in the dark or exposed to blue light illumination for the indicated times, before being subjected to Duolink PLA assay and analyzed by flow cytometry to quantitatively assess the interaction of endogenous SHANK3 and Rap1. Cells kept in the dark and untreated with primary antibodies served as controls. Data represent means±s.e.m. of five experiments (N.S., not significant; *P<0.05; paired two-tailed Student's *t*-test). Cells transduced with lentivirus encoding shRNA to knock down SHANK3 were also used as a further control to demonstrate the specificity of the PLA signal in four out of the five experiments (*P<0.05; unpaired two-tailed Student's *t*-test).

In response to blue light, CRY2–mCherry–talin interacts with the N terminus of CIBN–GFP–CAAX, thereby orienting the talin FERM domain along the plasma membrane while simultaneously interacting with two Rap1 molecules (Liao et al., 2021). In addition, binding of the FERM F2–F3 interface of talin with membrane lipids (Chinthalapudi et al., 2018) unravels the cloverleaf structure of talin, thereby weakening its autoinhibition (Dedden et al., 2019). To determine whether photo-enforced translocation of talin to the plasma membrane of endothelial cells might in fact liberate Rap1–GTP from SHANK3, the interaction between SHANK3 and Rap1 was examined using the PLA assay (Fig. 4A). The PLA signal representing the interaction between SHANK3 and Rap1 was observed throughout cellular compartments, including the cytoplasm and cell membrane (Fig. 4B; Fig. S4). To assess the interaction between SHANK3 and Rap1 following optogenetic translocation of talin to the plasma membrane, the fluorescent PLA signal was analyzed by flow cytometry. When the PLA signal was expressed as the fold increase over the signal in a sample omitting the primary PLA antibodies, it was approximately threefold higher than that observed in the same cells expressing SHANK3 shRNA (Fig. 4C, *P*<0.05). The interaction between SHANK3 and Rap1 gradually decreased following optogenetic translocation of talin to the plasma membrane, with an approximately 40% reduction in the interaction after 30 min of blue light illumination (Fig. 4C, *P*<0.05). Thus, translocation of talin to the endothelial cell plasma membrane appears to release Rap1–GTP from SHANK3 in approximately the same time interval that Rap1 activation is observed.

## DISCUSSION

An ‘inside-out’ integrin activation signaling cascade in cells eventually leads to the recruitment of the cytoskeletal protein talin to the plasma membrane. However, the precise mechanisms governing talin recruitment to the membrane appear to vary across integrin classes and cell types (Lagarrigue et al., 2015; Lee et al., 2009). In platelets and endothelial cells, evidence indicates that extracellular agonists lead to activation of membrane-anchored Rap1–GTP, which can then interact with the F0 and F1 subdomain of the talin head, thus modulating its positioning on the plasma membrane to interact with and activate integrins (Gingras et al., 2019; Zhu et al., 2017). In the present study, we identified an additional nuance to this canonical process: translocation of full-length talin to the plasma membrane can itself promote Rap1 activity through a process that relieves Rap1–GTP from its sequestration by SHANK3. Thus, talin in effect appears to function as both an activator and an effector of Rap1 in integrin signaling.

There is no evidence to date that talin regulates Rap1 at the level of a Rap1 guanine nucleotide exchange factor to convert inactive Rap1–GDP to active Rap1–GTP or to block the conversion of Rap1–GTP to inactive Rap1–GDP by a Rap1 guanine nucleotide-activating protein. Instead, based on previous work and analysis (Cai et al., 2020; Lilja et al., 2017; Atherton and Ballestrem, 2017), we focused on the possibility that talin liberates Rap1–GTP from SHANK3, a protein that can sequester Rap1–GTP with 1:2 stoichiometry. Notably, Rap1–GTP binding to SHANK3 is rather transient (Cai et al., 2020), potentially allowing for the dynamic regulation of Rap1 signaling by talin. Indeed, our findings are consistent with the idea that talin regulates Rap1 activity at the plasma membrane by competing with SHANK3 for interaction with Rap1–GTP.

The SPN domain of SHANK3 bears a structural similarity to a ubiquitin-like fold within the F0 subdomain of the talin FERM region, the latter being important for its interaction with Rap1 (Lilja et al., 2017). In unstimulated cells, cytoplasmic talin maintains an autoinhibited, inactive conformation with its FERM domain shielded by the rod domain (Dedden et al., 2019). Upon talin recruitment to the plasma membrane, this autoinhibition is relieved, likely in part through interaction with membrane phosphatidylinositol 4,5-bisphosphate (PIP_2_) (Litschel et al., 2024). Thus, talin interaction with the plasma membrane would expose its FERM domain, theoretically enabling it to compete with SHANK3 for binding to membrane-anchored Rap1–GTP.

The spatial localization of active Rap1 at cell edges and protrusions aligns with its known role in endothelial cell migration and angiogenesis. Our imaging data show that talin recruitment leads to Rap1 activation at critical cellular structures such as lamellipodia and pseudopodia (Fig. 2), thereby facilitating dynamic cellular responses such as migration. This is consistent with previous findings showing that light-induced talin recruitment to the plasma membrane promotes endothelial cell migration (Liao et al., 2021).

Cellular signal transduction pathways regulate levels of inactive Rap1–GDP and active Rap1–GTP through the action of guanine nucleotide exchange factors and GTPase-activating proteins (Bos et al., 2007). In addition to the known role of talin as a Rap1 effector for integrin activation in cells such as endothelial cells and platelets, the present study establishes that talin can also function to regulate the availability of functional Rap1–GTP at the plasma membrane, likely by competing with SHANK3. Given that SHANK3 is mutated in its SPN domain in a spectrum of neuropsychiatric disorders including autism spectrum disorder (Huang et al., 2023; Naisbitt et al., 1999), it might be of interest to further explore the Rap1–talin–integrin axis in neurons expressing WT and mutant SHANK3.

## MATERIALS AND METHODS

### Materials and DNA constructs

Alexa Fluor 647-conjugated fibrinogen was purchased from Thermo Fisher Scientific (Waltham, MA, USA). The mouse monoclonal antibody to Rap1 was from Proteintech (Rosemont, IL, USA; 67174). The rabbit polyclonal anti-DsRed antibody was from Takara (Mountain View, CA, USA; 632496). The mouse monoclonal anti-β-actin antibody was from Sigma-Aldrich (St Louis, MO, USA; A2228). The mouse monoclonal anti-GST antibody was from Santa Cruz Biotechnology (Santa Cruz, CA, USA; sc-138). The constructs encoding CRY2–mCherry–talin [containing the full-length sequence of mouse talin-1 (TLN1) in pcDNA3.1 Zeo(+) (Invitrogen)] and CIBN–GFP–CAAX in pLVX lentivirus vector (Takara Bio) were generated as described previously (Liao et al., 2021). The sequence encoding mCherry–talin-1 without CRY2 was cloned into pcDNA3.1 Zeo(+) with a start codon to generate the mCherry–talin construct. Mutations in CRY2–mCherry–talin were created using the KAPA HiFi kit (Kapa Biosystems, Wilmington, MA, USA). Lentiviral constructs in the FG12 vector (kindly provided by Roger Tsien, University of California, San Diego) encoding shRNAs were generated as described previously (Kato et al., 2012). The target sequences for mouse SHANK3 shRNAs were: shRNA-1, 5′-GGAAGTCACCAGAGGACAAGA-3′, and shRNA-2, 5′-CCGATACAAGCGGAGAGTTTA-3′. To generate SHANK3–Myc–mAzurite, fragments encoding mAzurite and SHANK3–Myc were amplified from the plasmids encoding mAzurite–actin (Addgene, plasmid #55227) and Myc-tagged rat SHANK3 (a kind gift from Dr Hans-Jürgen Kreienkamp, University Medical Center Hamburg-Eppendorf), and ligated and cloned into pcDNA3.1 hygro(+) (Invitrogen). PCR-mediated mutagenesis was used to delete SHANK3 from SHANK3–Myc–mAzurite to generate the Myc–mAzurite construct in pcDNA3.1 hygro(+). Similarly, the DNA fragments encoding mAzurite and the FLAG-tagged SPN domain of SHANK3 (Lilja et al., 2017) were amplified, ligated and cloned into the pLVX-IRES-hygro vector (Takara Bio).

### Cell culture and transfection

Immortalized murine lung endothelial cells that stably express CRY2–mCherry–talin were generated using CRISPR/Cas9-mediated homology-directed repair as described previously (Liao et al., 2021). The cells were then transduced with the lentivirus encoding CIBN–GFP–CAAX. A5 CHO cells that stably express CRY2–mCherry–talin and CIBN–GFP–CAAX were created previously (Liao et al., 2021). Immortalized murine endothelial cells and A5 CHO cells were cultured in Dulbecco's modified Eagle medium (DMEM; Gibco) supplemented with non-essential amino acids, antibiotics, L-glutamine and 10% fetal bovine serum (Cell Culture Collective), with or without 10 ng/ml recombinant VEGF (Biolegend) as indicated.

### Western blotting

Cells were lysed in NP-40 buffer [1% Nonidet P-40, 150 mM NaCl, 50 mM Tris HCl, pH 8.0, and EDTA-free complete protease inhibitor cocktail (Roche Applied Science)]. Proteins were resolved by SDS-PAGE and transferred to nitrocellulose membranes. After blocking with 5% bovine serum albumin in Tris-buffered normal saline, membranes were incubated with the following primary antibodies: anti-Rap1 (Proteintech, 67174; 1:500 dilution), anti-talin (Millipore-Sigma, T3287; 1:1000 dilution), anti-β-actin (Sigma-Aldrich, A2228; 1:2000 dilution), anti-Myc (Santa Cruz Biotechnology, sc-40; 1:500 dilution), anti-FLAG (Millipore-Sigma, F3165; 1:1000 dilution) and anti-SHANK3 (Cell Signaling Technology, 64555; 1:1000 dilution). Membranes were then incubated with secondary antibodies conjugated to IRDye 800CW or IRDye 680RD (LI-COR Biosciences, 925-32211 and 925-68072; 1:10,000 dilution). Western blots were analyzed with the Odyssey imaging system (LI-COR Biosciences) and the raw western blot data are provided in Fig. S5 as ‘Blot transparency’.

### Rap1 activation

Rap1 activity was detected using the RBD of human RalGDS fused to GST as described previously (Schmitt and Stork, 2002). Briefly, the RalGDS RBD–GST fusion protein expressed in *Escherichia coli* was bound to glutathione Sepharose-4B beads (Amersham Biosciences). To examine optogenetic Rap1 activity, cells suspended in serum-free DMEM were illuminated using blue LED light (1 s illumination per 75 s; 50 mW/cm^2^) for the indicated time at room temperature and then lysed using 2× NP-40 buffer [2% Nonidet P-40, 300 mM NaCl, 100 mM Tris-HCl, pH 8.0, 2× phosSTOP (Roche Applied Science) containing 2× EDTA-free complete protease inhibitor cocktail (Roche Applied Science)]. Samples with equal amounts of total protein were incubated with 25 µl of RalGDS RBD–GST bead slurry at 4°C for 2 h and then submitted to electrophoresis on 4–20% gradient SDS-PAGE gels. Rap1 protein was then detected by immunoblotting with anti-Rap1 antibody (Proteintech, 67174; 1:500 dilution). Immortalized mouse endothelial cells were also treated with 100 ng/ml basic FGF (Millipore-Sigma, GF446-10UG) in the dark for the indicated time before the Rap1 activation assay.

### *In situ* Rap1–GTP detection

To examine local Rap1 activity in cells, the purified GST–RalGDS fusion protein was used as previously described (Taniguchi et al., 2008). Briefly, immortalized mouse endothelial cells expressing CIBN–GFP–CAAX and CRY2–mCherry–talin were either suspended in 500 μl Hank's balanced salt solution (HBSS; Gibco) containing 1% bovine serum albumin or plated onto fibronectin-coated coverslips for 30 min before being illuminated using blue LED light for 30 min at room temperature. Cells were then fixed with 3.7% formaldehyde, permeabilized with 0.1% Triton X-100, and blocked with 10% goat serum for 1 h before overnight incubation with GST–RalGDS (6 µg/ml) at 4°C. After washing, cells were fixed with 2% formaldehyde for 10 min and then incubated with a 1:200 dilution of mouse anti-GST monoclonal antibody (Santa Cruz Biotechnology, sc-138) and rabbit anti-DsRed antibody (Takara Bio, 632496) for 2 h at room temperature. Alexa Fluor 647-conjugated anti-mouse and Alexa Fluor 546-conjugated anti-rabbit antibodies (Invitrogen, A-21236 and A-11010; 1:500 dilution) were used as secondary antibodies. Native GFP fluorescence was used to detect the localization of CIBN–GFP–CAAX. Cells in suspension or on coverslips were mounted on slides using the ProLong Diamond antifade reagent (Thermo Fisher Scientific). Images were taken using a Zeiss Elyra 7 Lattice structured illumination microscope with a 100× oil objective. For cells adherent to fibronectin-coated coverslips, a total internal reflection fluorescence (TIRF) microscopy setup was used. Briefly, in the laser imaging mode, observation of samples can be switched between EPI and TIRF mode. TIRF angle was calibrated using TIRF beads after installation of the software Zen. Focus of the sample was achieved in EPI mode then switched to TIRF mode. The TIRF angle slider was used to optimize the best TIRF setting.

### Duolink PLA

The Duolink PLA kit for flow cytometry with mouse/rabbit probes and far-red detection (Millipore-Sigma, DUO94104) was used for PLA. Briefly, immortalized mouse endothelial cells (1×10^7^) expressing CIBN–GFP–CAAX and CRY2–mCherry–talin were illuminated with blue LED light for the indicated time, fixed, permeabilized and stained overnight at 4°C with a pair of antibodies [rabbit anti-mCherry (Takara Bio, 632496; 1:100 dilution) and mouse anti-Rap1 (Proteintech, 67174; 1:100 dilution) for Fig. 3G; or rabbit anti-SHANK3 (Cell Signaling Technology, 64555; 1:100 dilution) and mouse anti-Rap1 for Fig. 4]. Incubation with secondary anti-mouse MINUS and anti-rabbit PLUS probes (Millipore-Sigma), ligation and amplification were carried out in solution according to the manufacturer's instructions. The PLA signal represented by the mean fluorescence intensity was detected using an Accuri C6 Plus flow cytometer (BD Biosciences) after incubation with Far-Red detection solution (Millipore-Sigma) for 1 h at 37°C. Samples without primary antibody were used as a negative control. The Duolink PLA assay was also performed on cells adherent to chamber slides to visualize an *in situ* PLA signal. Coverslips were mounted on slides using the ProLong Diamond antifade reagent. Images were taken with an Olympus IX81 inverted microscope using a 60× oil objective.

### Integrin activation

Integrin αVβ3 activation in immortalized murine endothelial cells was assessed by flow cytometry with Alexa Fluor 647-conjugated fibrinogen. Briefly, (4–6)×10^5^ cells in 50 μl HBSS containing 1% bovine serum albumin were incubated with Alexa Fluor 647-conjugated fibrinogen and illuminated using blue LED light for 30 min at room temperature before fixation with 3.7% formaldehyde. Nonspecific fibrinogen binding was determined in the presence of 5 mM EDTA, and specific binding was taken as the total binding minus nonspecific binding.

Integrin αIIbβ3 activation in A5 CHO cells was assessed by flow cytometry with Alexa Fluor 647-conjugated antibody PAC-1 (Biolegend, 362806). Briefly, 50 μl of cell suspension was incubated with 2 μl of Alexa Fluor 647-conjugated PAC-1 and illuminated using blue LED light for 30 min at room temperature before fixation with 3.7% formaldehyde. Nonspecific PAC-1 binding was determined in the presence of 5 mM EDTA, and specific binding was taken as the total binding minus nonspecific binding.

### Statistical analysis

A paired, two-tailed Student's *t*-test was used to calculate differences between two groups. *P*-values of ≤0.05 were taken as statistically significant.

## Supplementary Material

10.1242/joces.263595_sup1 Supplementary information
