# Supplementary material for: Talin, a Rap1 effector for integrin activation at the plasma membrane, also promotes Rap1 activity by disrupting sequestration of Rap1 by SHANK3
Source: J Cell Sci. 2025 Feb 26;138(4):JCS263595. doi: 10.1242/jcs.263595 (PMC11928058; doi:10.1242/jcs.263595)
Supplement: Supplementary information [file joces-138-263595-s1.pdf]

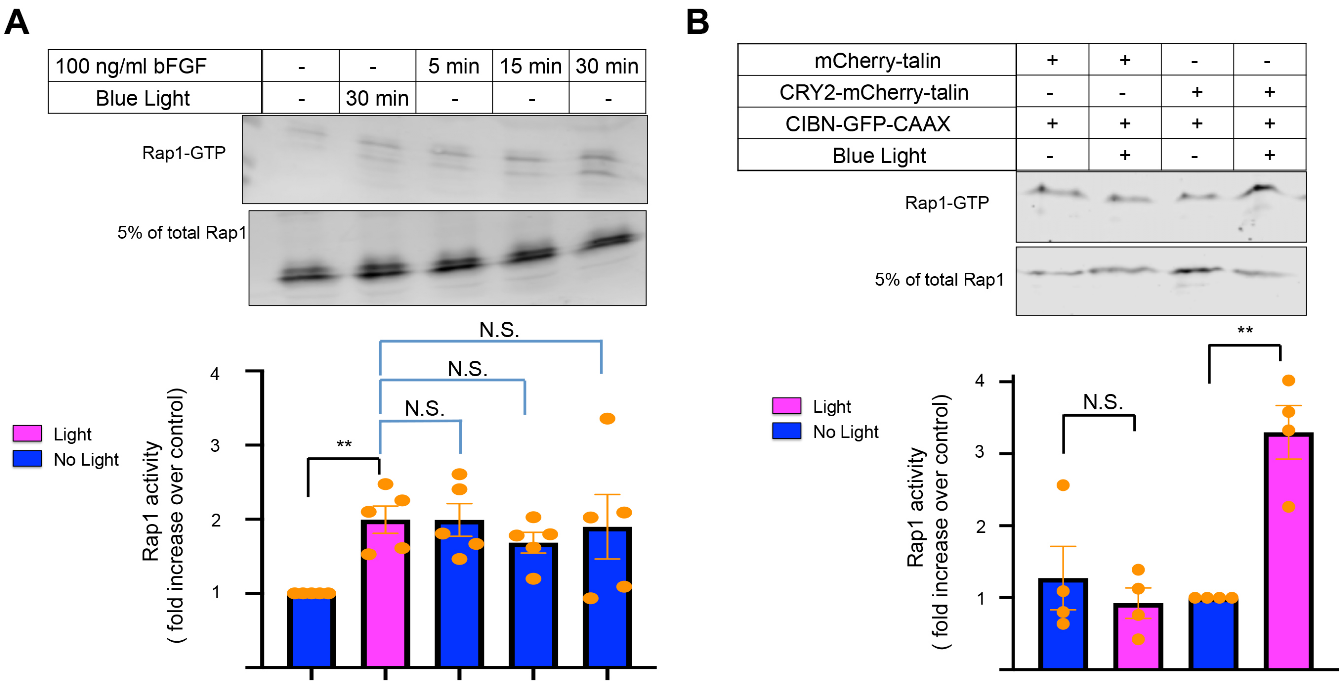


**Fig. S1.** Rap1 activation after optogenetic recruitment of talin to the plasma membrane. (*A*) Immortalized mouse lung endothelial cells expressing CIBN-GFP-CAAX and CRY2-mCherry-talin were exposed to either blue light illumination or 100 ng/ml bFGF for the indicated times prior to measurement of Rap1-GTP. Untreated cells kept in the dark served as a control. Data represent means ± SEM of five experiments (double asterisk, $p < 0.01$; N.S., not significant). (*B*) No Rap1 activation was observed in cells expressing CIBN-GFP-CAAX and mCherry-talin (without CRY2). Data represent means ± SEM of four experiments (double asterisk, $p < 0.01$; N.S., not significant).

Journal of Cell Science • Supplementary information

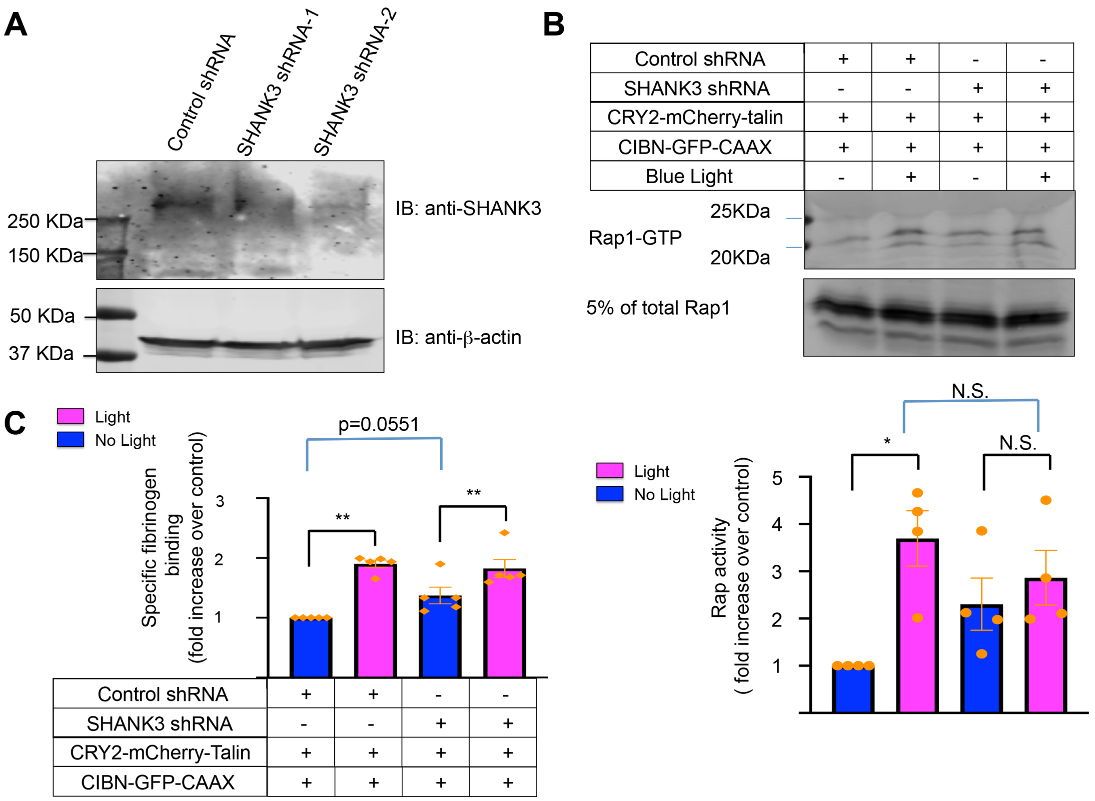


**Fig. S2.** Impact of SHANK3 knockdown on optogenetic activation of Rap1. (*A*) Immortalized mouse lung endothelial cells expressing CIBN-GFP-CAAX and CRY2-mCherry-talin were transduced with lentiviruses encoding either of two SHANK3 shRNAs or control shRNA. SHANK3 expression was assessed by Western blot. β-actin served as a loading control. SHANK3 shRNA-2 was used for the following assays. (*B*) Rap1 activity was determined and expressed as the fold increase of Rap-GTP relative to that observed when the cells were maintained in the dark. Data represent means ± SEM of four experiments (asterisk, $p < 0.05$; N.S., not significant). (*C*) Specific fibrinogen binding to endothelial αVβ3 following optogenetic recruitment of talin to the plasma membrane in cells expressing SHANK3 shRNA or control shRNA. Data represent means ± SEM of five experiments (double asterisk, $p < 0.01$).

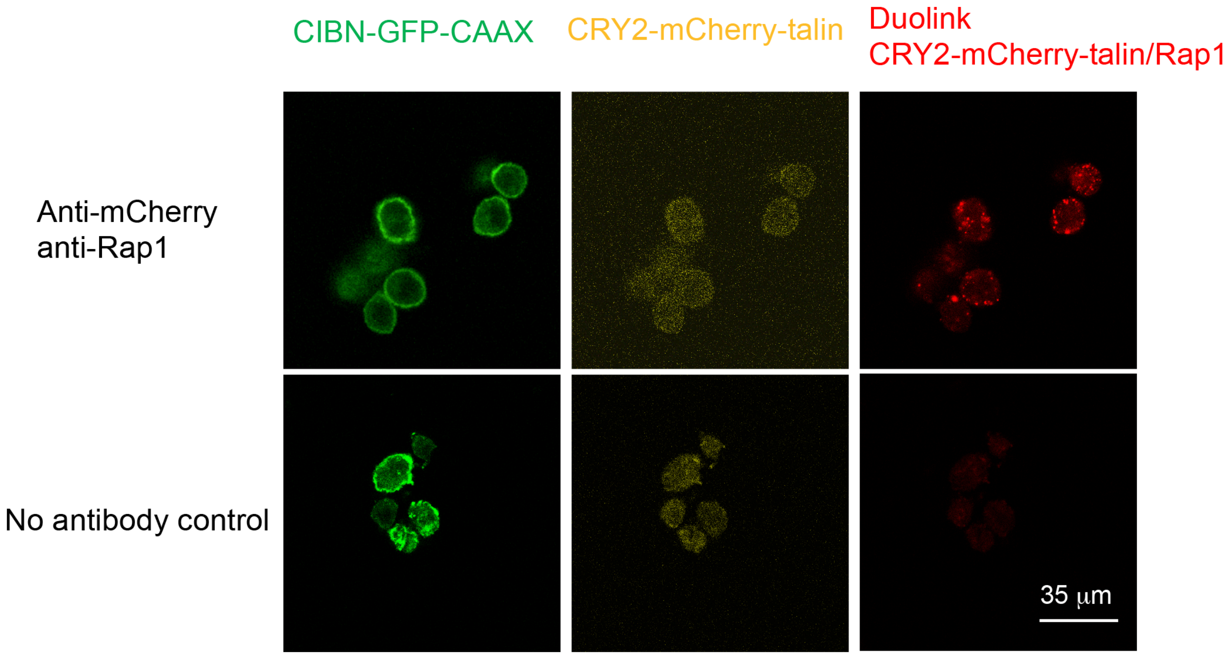


**Fig. S3.** Direct interaction between CRY2-mCherry-talin and Rap1 detected by Duolink PLA *in situ* assay. Immortalized murine lung endothelial cells expressing CRY2-mCherry-talin (middle panels) and CIBN-GFP-CAAX (left panels) were fixed, permeabilized and stained with antibodies to mCherry and Rap1. Then proximity ligation assay was performed to assess the extent of co-localization of CRY2-mCherry-talin and endogenous Rap1 (right panels). Cells were imaged by confocal microscopy. The proximity ligation signal (right upper panel) was observed predominantly at the plasma membrane (localized by CIBN-GFP-CAAX). In the lower panels, cells kept in the dark and untreated with primary antibodies were used as controls. Scale bar, 35 μm.

Journal of Cell Science • Supplementary information

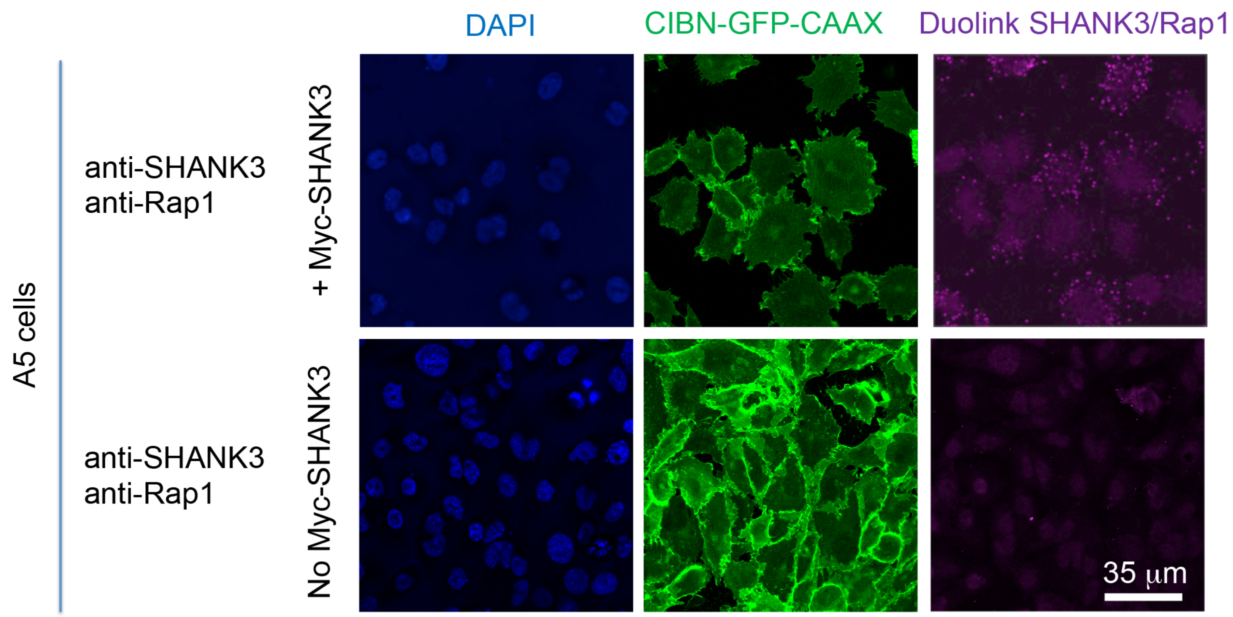


**Fig. S4.** Proximal interaction between SHANK3 and Rap1. *In situ* Duolink proximity ligation assay was performed in A5 CHO cells expressing CIBN-GFP-CAAX and CRY2-mCherry-talin to assess a potential proximal interaction between SHANK3 and Rap1. Cells expressing Myc-SHANK3 (upper panels) or no Myc-SHANK3 (lower panels) were cultured overnight, fixed, permeabilized, and stained with anti-SHANK3 and anti-Rap1 antibodies. Proximity ligation assay was performed to evaluate co-localization of SHANK3 and Rap1. Cell nuclei were counterstained with DAPI and cells imaged by confocal microscopy. Scale bar, 35 μm.

Journal of Cell Science • Supplementary information

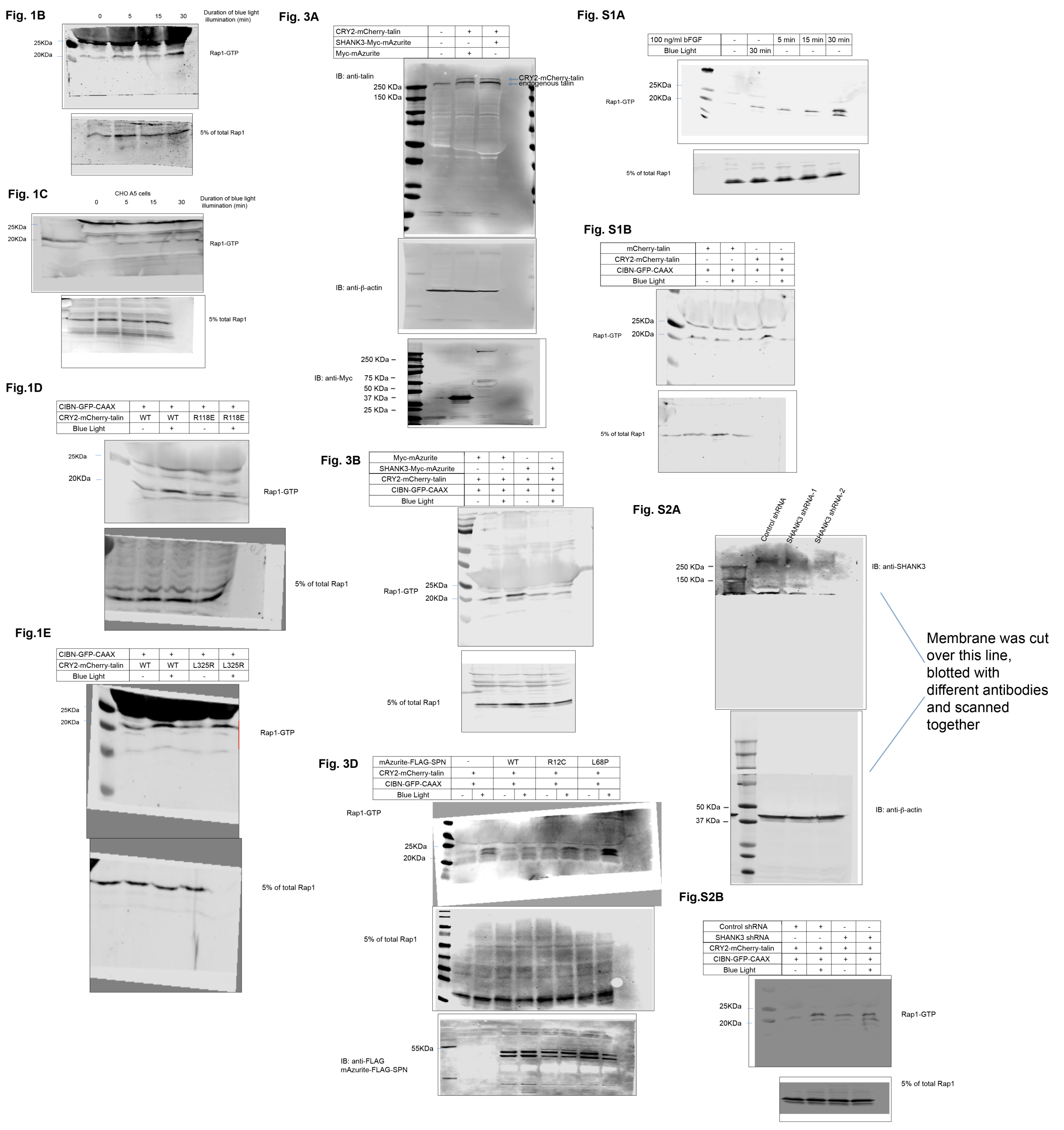


**Fig. S5.** Blot transparency. The molecular weights of the protein ladder are labeled.

Journal of Cell Science • Supplementary information
